# A 660-Kb Deletion with Antagonistic Effects on Fertility and Milk Production Segregates at High Frequency in Nordic Red Cattle: Additional Evidence for the Common Occurrence of Balancing Selection in Livestock

**DOI:** 10.1371/journal.pgen.1004049

**Published:** 2014-01-02

**Authors:** Naveen Kumar Kadri, Goutam Sahana, Carole Charlier, Terhi Iso-Touru, Bernt Guldbrandtsen, Latifa Karim, Ulrik Sander Nielsen, Frank Panitz, Gert Pedersen Aamand, Nina Schulman, Michel Georges, Johanna Vilkki, Mogens Sandø Lund, Tom Druet

**Affiliations:** 1 Center for Quantitative Genetics and Genomics, Department of Molecular Biology and Genetics, Aarhus University, Tjele, Denmark; 2 Unit of Animal Genomics, GIGA-R & Faculty of Veterinary Medicine, University of Liège (B34), Liège, Belgium; 3 MTT Agrifood Research Finland, Biotechnology and Food Research, Jokioinen, Finland; 4 Danish Agricultural Advisory Service, Aarhus N, Denmark; 5 Molecular Genetics and Systems Biology, Department of Molecular Biology and Genetics, Aarhus University, Tjele, Denmark; 6 Nordic Cattle Genetic Evaluation, Aarhus N, Denmark; University of Bern, Switzerland

## Abstract

In dairy cattle, the widespread use of artificial insemination has resulted in increased selection intensity, which has led to spectacular increase in productivity. However, cow fertility has concomitantly severely declined. It is generally assumed that this reduction is primarily due to the negative energy balance of high-producing cows at the peak of lactation. We herein describe the fine-mapping of a major fertility QTL in Nordic Red cattle, and identify a 660-kb deletion encompassing four genes as the causative variant. We show that the deletion is a recessive embryonically lethal mutation. This probably results from the loss of *RNASEH2B*, which is known to cause embryonic death in mice. Despite its dramatic effect on fertility, 13%, 23% and 32% of the animals carry the deletion in Danish, Swedish and Finnish Red Cattle, respectively. To explain this, we searched for favorable effects on other traits and found that the deletion has strong positive effects on milk yield. This study demonstrates that embryonic lethal mutations account for a non-negligible fraction of the decline in fertility of domestic cattle, and that associated positive effects on milk yield may account for part of the negative genetic correlation. Our study adds to the evidence that structural variants contribute to animal phenotypic variation, and that balancing selection might be more common in livestock species than previously appreciated.

## Author Summary

We report the identification of a large deletion encompassing four genes and the demonstration of its negative effect on fertility in Nordic Red dairy cattle. We show that this deletion is recessively lethal (homozygous embryos die) and therefore, when carrier cows are mated to carrier bulls, there is a high risk of embryonic mortality. As a result, chances of insemination failure are higher for such matings. Surprisingly, despite its negative effect, the deletion is frequent in Nordic Red cattle. We show that this high frequency may be a consequence of the fact that the deletion is associated with increased milk production and therefore selected for. Due to increased levels of inbreeding resulting from the widespread use of artificial insemination, such recessive lethal alleles may account for a non-negligible fraction of the reduction in fertility observed in cattle.

## Introduction

Widespread application of artificial insemination, combined with the use of the animal model (exploiting kinship inferred from pedigree and/or genome-wide SNP data) to accurately predict breeding values, has led to spectacular increases in the productivity of livestock. As an example, average milk yield per lactation has nearly doubled in US Holstein cows between 1960 (∼6,300 kgs) and 2000 (∼11,800 kgs), and more than half of this progress was genetic [1]. Milk yield and composition have moderate heritability (20–40%), and – with the exception of a handful of genes with detectable effects including *DGAT1* [2]- their genetic architecture is quasi-infinitesimal (e.g., [3], [4]).

During the same period, cow fertility has declined severely in most countries. In the same US dairy cattle population, the number of days between calving and first estrus has increased from 126 to 169 between 1976 and 1999 [5]. Lucy [6] reports that, between 1970 to 2000, the number of inseminations required to obtain a pregnancy increased from 1.8 to 3.0, and that the interval between successive calvings increased from 13.5 to 14.9 months in US Holstein. Between 1972 and 1996, the conception rate for the first insemination reportedly dropped from 62% to 34% [7]. Fertility traits in cattle have low heritability, ranging from 1 to 10% (e.g., [8]).

Fertility is negatively correlated with milk yield and composition. For example, the genetic correlation between milk yield and interval between calving and first insemination is 0.43 (e.g., [8]). It is generally assumed that the reduction in fertility is due to the negative energy balance of high-producing cows at the peak of lactation (e.g., [6]). The genetic architecture of fertility is consequently assumed to be primarily quasi-infinitesimal as well (e.g., [4]).

It was recently observed that the typical human carries >100 loss-of-function (LoF) variants [9]. Epidemiological evidence indicates that a handful of these might be highly deleterious in homozygotes, including by causing embryonic death [10]. It was recently shown that the majority of conceptuses that are homozygous for LoF mutations in the bovine *SLC35A3* and *FANCI* genes causing complex vertebral malformation and brachyspina, respectively, die before birth [11], [12]. The main economic impact of these genetic defects might thus result from their effect on fertility rather than from calf mortality *per se*. The observation of significant depletions in autozygosity for specific haplotypes suggests that several other embryonically lethal mutations (EL) are segregating at intermediate frequencies in livestock populations and jointly account for a non-negligible proportion of insemination failures [13]–[15].

We herein report the positional cloning of a quantitative trait locus (QTL) with major effect on cow fertility. We show (i) that the causative mutation is a 660-Kb deletion that encompasses four genes on bovine chromosome 12 (*Bos taurus* – BTA12), (ii) that it affects fertility by causing early embryonic death of homozygous conceptuses, and (iii) that it is maintained at high frequency in Nordic Red breeds because of its association with positive effects on milk yield and composition. Our results thus add to the evidence that the spread of recessive embryonic lethal variants account for at least part of the decline in fertility observed in cattle. This is at least the seventh example in livestock where an allele that is deleterious at the homozygous state is maintained at high frequency in the population because of the selective advantage it confers to heterozygotes.

## Results

### A QTL with major effect on cow fertility maps to BTA12

**Figure 1:**
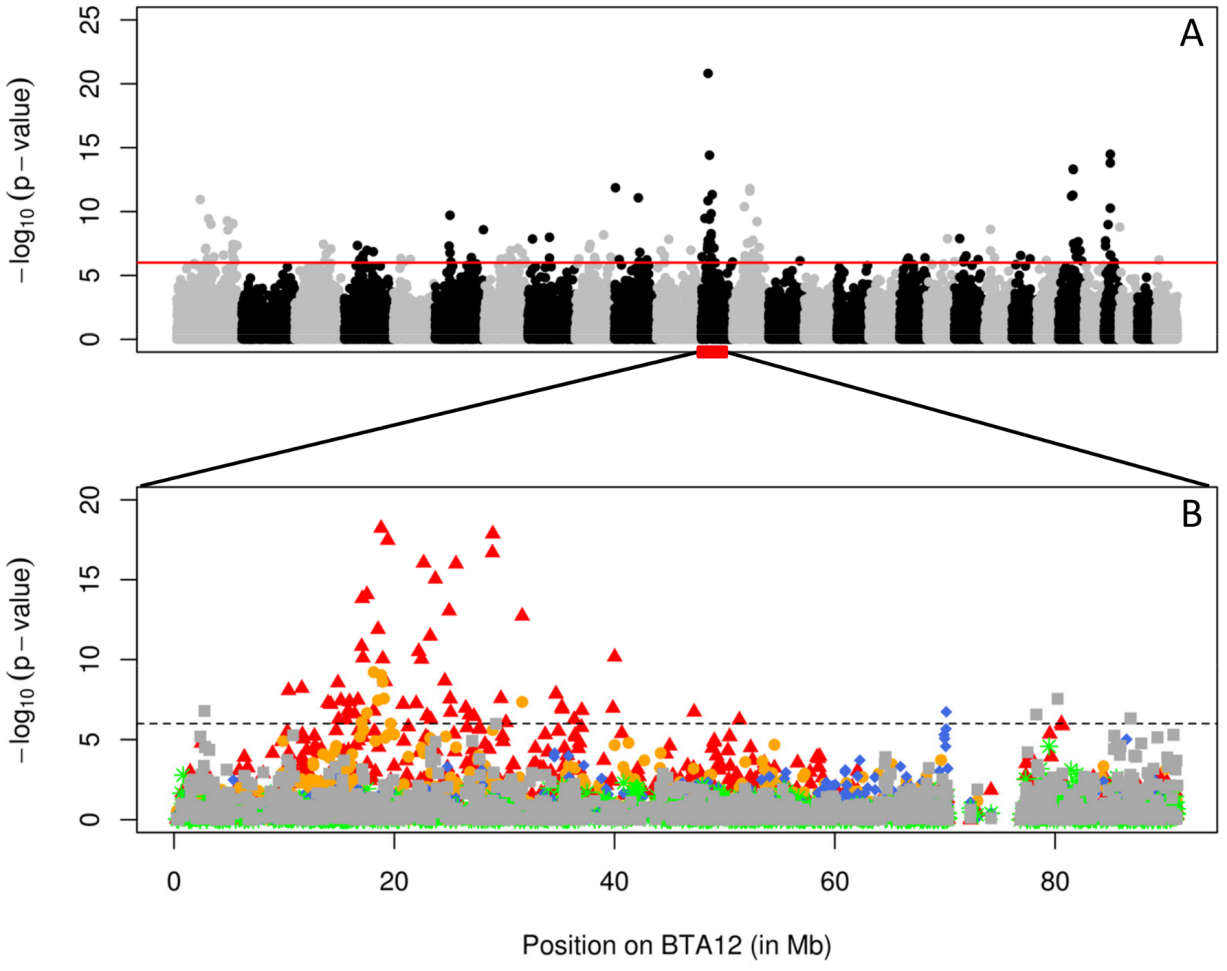
Association scan of the bovine genome for QTL influencing fertility. A. Genome-wide Manhattan plot obtained by across-breed single-point analysis for an index combining fertility traits (number of inseminations in heifers and cows (AISH and AISC), interval between calving and first insemination (ICF) and interval between first and last insemination in heifers and cows (IFLH and IFLC)). B. Chromosome-specific (BTA12) plots corresponding to within-breed single-point analyses for the same fertility index (red: Finnish Ayrshire, orange: Swedish red, blue: Danish red, green: Jersey and gray: Holstein cattle). The horizontal lines mark the genome-wide significance threshold.

To map QTL influencing cow fertility, we performed a genome wide association study (GWAS) using a cohort comprising 4,072 Holstein-Friesian, 1,177 Jersey, 894 Danish Red, 1,714 Swedish Red, and 2,242 Finnish Ayrshire progeny-tested bulls. The 10,099 bulls were genotyped using the 50K Bovine Array (Illumina, San Diego, CA). The phenotypes for this initial scan were the bull's predicted breeding values (EBV) for an index combining the different fertility traits (number of inseminations in heifers and cows (AISH and AISC), interval between calving and first insemination (ICF) and interval between first and last insemination in heifers and cows (IFLH and IFLC)). Association analysis was conducted using all animals on a SNP-by-SNP basis (assuming an additive model), yet accounting for familial relationships and population stratification by including a random sire effect and four principal components. We obtained 14 genome-wide significant QTL, of which one on chromosome 12 was the strongest (p<10^−20^; Figure 1A). We repeated the analysis of BTA12 by breed, and this indicated that the QTL was mainly segregating in the Finnish Ayrshire and Swedish Red, but was not detectable in Holstein-Friesian, Danish Red and Jerseys (Figure 1B). A QTL influencing fertility has previously been reported at approximately the same position in Finnish Ayrshire [16], [17] and Norwegian Red [18].

### The QTL is entirely explained by a 660-Kb deletion with negative effect on fertility

We repeated the association analysis using a previously described haplotype-based method including an animal model [19], in Finnish Ayrshire and Swedish Red. Haplotyping was done jointly across all Nordic red breeds, while the association analysis was conducted separately within each breed. We now analyzed fertility traits individually, including number of inseminations for cows/heifers (AISC & AISH), interval between calving and first insemination (ICF), interval between first and last insemination for cows/heifers (IFLC & IFLH), non-return (to heat after insemination) rate at 56 days for cows/heifers (NRRC & NRRH), and heat strength (HS). Genome-wide significant signals were obtained in both breeds at the expected map position for all tested traits except ICF and HS (Figure S1). It is worth noting that ICF and HS are related to oestrus while all other traits are related to pregnancy success. One of the 40 fitted ancestral haplotypes (hereafter called haplotype A27 – see Figure S2), shared across breeds, was shown to have a pronounced negative effect on all fertility traits affected by the QTL (Figure S1A–G).

Closer examination of the SNPs in Finnish Ayrshire in the immediate vicinity of the association peak identified five markers that departed very significantly from Hardy-Weinberg equilibrium (p-values ranging from 10^−66^ to 10^−161^) as a result of excess homozygosity (Figure S3A). The same markers were also characterized by an inflation of Mendelian parent-offspring incompatibilities (Figure S3B). Both findings suggested the occurrence of a chromosomal deletion.

**Figure 2:**
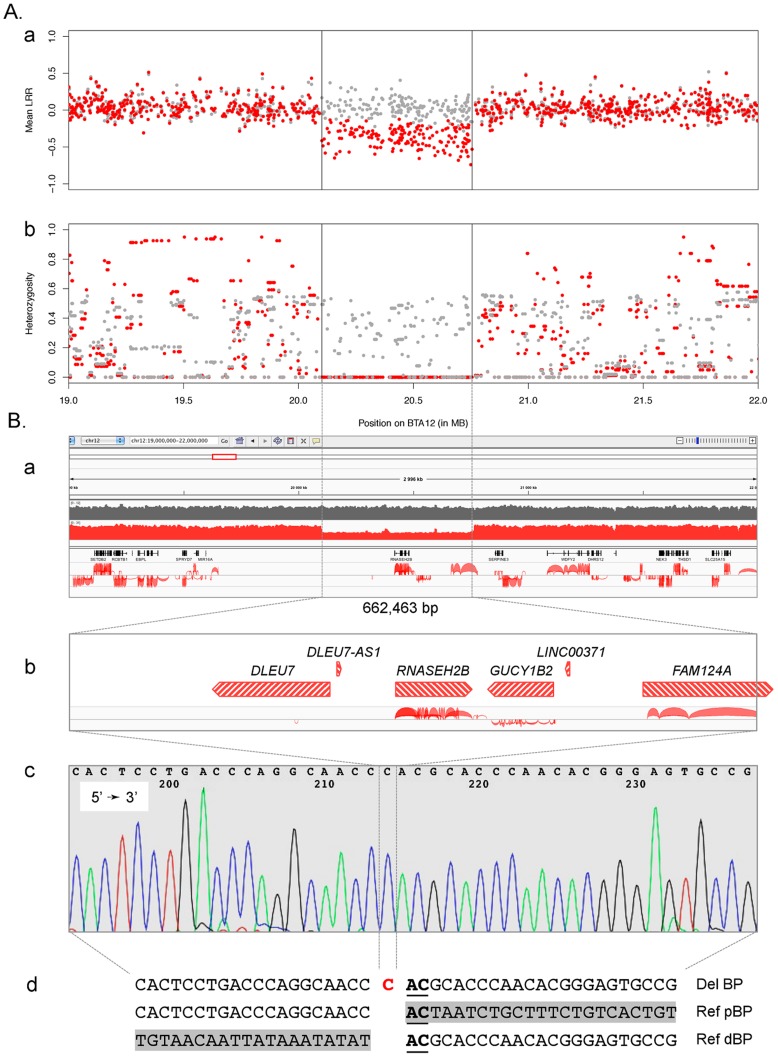
Characterization of the BTA12 deletion. A. Characterization of the deletion using SNPs from the BovineHD genotyping array (Illumina, San Diego, CA): (a) average signal intensity (LRR) and (b) mean heterozygosity per marker for carriers (red) and non-carriers (gray). B. Characterization of the deletion at the sequence level: (a) Integrative Genomics Viewer (IGV – http://www.broadinstitute.org/igv/) screen capture of NGS data featuring the 3 Mb region encompassing the deletion with, from top to bottom, tracks of depth coverage for a wild-type (gray) and a carrier (red) animal, a track of RefSeq gene annotation and a RNA-seq junctions output track obtained with *TopHat* [44]; (b) zoom of the gene content within the 660 kb deletion, where transcriptional units are depicted as transcription-oriented hatched red arrows above RNA-seq data; (c) sequence trace of the 281 bp amplicon bridging the breakpoint (BP); (d) alignment of deleted sequence (Del BP) with wild-type proximal (Ref pBP) and distal (Ref dBP) BP sequences highlighting the “C” insertion (red) and the 2 bp microhomology (underlined); gray boxes correspond to wild-type sequences within the deletion, directly adjacent to the respective BP boundaries.

To test this hypothesis, we first took advantage of available SNP genotypes obtained with the 770K HD bovine array (Illumina) for 243 Finnish Ayrshire (including 82 animals carrying haplotype A27) to search for structural variation. The animals carrying haplotype A27 were shown to present both reduced total signal intensity (referred to as “Log R ratio”) and complete homozygosity for 174 consecutive SNPs spanning positions 20,101,696 to 20,755,193, confirming the deletion hypothesis (Figure 2A). The same deletion was previously reported in a multi-breed CNV scan, in which it was only observed in Norwegian Reds [20].

We then took advantage of whole genome next generation sequencing (NGS) information available for 30 Red Danish and 18 Finnish Ayrshire bulls including respectively one and six individuals carrying haplotype A27. In all carrier animals, the occurrence of a deletion was obvious from (i) the approximately halved sequence depth from positions 20.10 to 20.76 Mb and (ii) the incongruent mapping of paired-ends separated by approximately 660 Kb (Figure 2B & Figure S4). In addition to the paired reads bridging the breakpoint, detailed analysis of individual sequences identified several split reads that sized the deletion at exactly 662,463 bp (position 20,100,649 to 20,763,116 bp) (Figure S5A). Proximal and distal breakpoints mapped to non-homologous LINE repeats (L1ME1 and L1BT respectively) and are characterized by 2-bp microhomology, while the deletion event was accompanied by a one bp insertion (Figure S2B). The breakpoint was confirmed by PCR amplification, using a forward primer in a unique sequence upstream of the L1ME1 repeat and a reverse primer within the L1BT element. The expected 281 bp PCR product was obtained from carriers, but not from homozygous wild-type controls. In comparison, an amplicon positioned within the deletion yielded the expected 318 bp product in the four animals (Figure S5B). Sanger sequencing of the 281 bp deletion-specific amplicon confirmed the NGS results (Figure 2B). Examination of the annotation of the orthologous region in mammals suggests that the deletion encompasses three protein-encoding genes (*RNASEH2B*, *GUCY1B2* and 3 out of 4 exons of *FAM124A*), one gene with uncertain coding potential (*DLEU7*) and two non-coding RNA genes (*DLEU7-AS1* and *LINC00371*) (Figure 2B). Whole-genome RNA-Seq reads available from the cortex of a 60 days post-fertilization bovine embryo, supported the organization and coding potential of the three protein coding genes, revealed reads mapping to the putative *DLEU7* gene, but no reads corresponding to the two putative non-coding RNA genes (Figure 2B).

**Figure 3:**
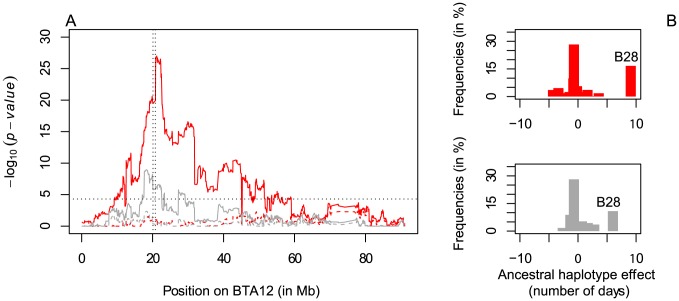
Fine-mapping of the fertility QTL on BTA12. A. Haplotyped-based QTL fine-mapping results on BTA12 for the trait “interval from first to last insemination of cows (IFLC)” in Finnish Ayrshire (red) and Swedish Red (gray) cattle. The x-axis represents the physical position on BTA12 and the y-axis the p-value of the likelihood ratio test. Full and dashed lines represent QTL mapping with and without correction for haplotype B28 tagging the deletion. The vertical dotted lines indicate the position of the deletion whereas the horizontal line marks the chromosome-wide significance threshold. B. Effect (in days) and frequency of the 40 ancestral haplotypes in Finnish Ayrshire (red) and Swedish Red (gray) cattle.

The 660-Kb deletion spans five SNPs interrogated by the 50K Bovine array. As the deletion might have compromised the phasing accuracy and hence the mapping accuracy, we first rephased SNP data after exclusion of the five corresponding SNPs and repeated the haplotype-based analysis described above. We obtained a chromosome-wide significant signal immediately adjacent to the 660-Kb deletion. It was entirely driven by one of the 40 newly fitted ancestral haplotypes (hereafter called haplotype B28 – see Figure S2), which had strong negative effect on fertility. Indeed, adding B28 genotype to the model completely annihilated the QTL signal (Figure 3A–B and Figure S6A–G). As expected, haplotype states B28 and A27 were closely related in the immediate vicinity of the deletion (Figure S2). Carriers of the B28 haplotype had a frequency of ∼32% in Finnish Ayrshire, ∼23% in Swedish Red and ∼13% in Danish Red (yet were absent in Holstein-Friesian and Jerseys). We then exploited signal intensity, obligate homozygosity and parentage conflicts for the corresponding markers to confidently genotype 2,139 Finnish Ayrshire, 1,221 Swedish Red and 1,096 Danish Red sires for the deletion (see Text S1). Linkage disequilibrium (r^2^) between the deletion and haplotype B28 was 0.96, indicating that haplotype B28 tagged the deletion nearly perfectly (see Text S1). Taken together, these findings indicate that the 660-Kb deletion is most likely the causative variant underlying the fertility QTL.

### Homozygosity for the 660-Kb deletion is embryonically lethal

The complete ablation of four genes, including *RNASEH2B* known to cause embryonic lethality when knocked-out in the mouse [21], [22], suggested that the 660-Kb deletion might affect fertility by causing embryonic death in homozygotes. This would also be compatible with the fact that the QTL affects the interval between first and successful insemination, number of inseminations and non-return rate (all related to pregnancy success), but not the interval from calving to first insemination and heat strength (related to oestrus). The EL hypothesis makes two predictions: (i) there should be no homozygotes for the 660-Kb deletion amongst live animals, and (ii) the fertility problems should be restricted to matings between carrier sires and carrier dams.

To test the first prediction we first examined the signal intensities for the five SNPs (50K array) within the deletion in 3,095 Finnish Ayrshires and 1,312 Swedish Red. The lowest average logRR value was −1.09, which is within the range of values expected for heterozygotes. As much lower values are expected for homozygotes, we can confidently conclude that none of these were present in the analyzed sample.

**Figure 4:**
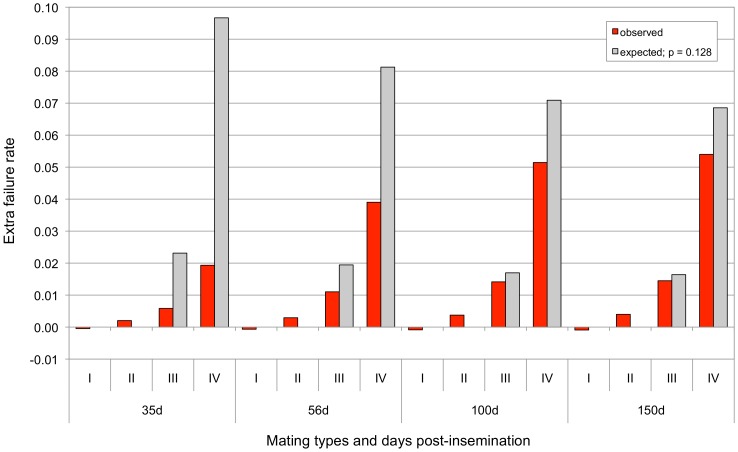
Increased reproductive failure rate in mating types sorted according the genotype of sire and maternal grand-sire for the 660 Kb deletion (I for non-carrier x non-carrier, II for non-carrier x carrier, III for carrier x non-carrier and IV for carrier x carrier matings defined as in the main text), at 35, 56, 100 and 150 days post-insemination. The default level of reproductive failure was set as the average level observed for mating types I and II (as no offspring homozygous for the deletion can be produced in these matings). The excess failure rates that were observed in the different mating types and at the different time-points are shown by the red bars. The excess failure rates that are expected assuming no development of homozygous conceptuses (calculated as described in the text and assuming a frequency of 0.128 for the deletion) are shown by the gray bars.

To test the second prediction, we compiled the rate of reproductive failure established by the fact that the cows returned in oestrus 35, 56, 100 and 150 days after insemination for matings sorted by genotype (sire and maternal grand-sire) for the 660-Kb deletion: (i) non-carrier (NC) sire X daughter of NC maternal grand-sire, (ii) NC sire X daughter of carrier (C) maternal grand-sire, (iii) C sire X daughter of NC maternal grand-sire, and (iv) C sire X daughter of C maternal grand-sire. The corresponding rates were estimated using a mixed model that included parity and month of insemination as fixed effects, and maternal grand-sire as random effect. The expected proportions of conceptuses that are homozygous for the 660-Kb deletion are, respectively, (i) 0, (ii) 0, (iii) 0.25*p*, and (iv) 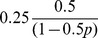 for the four different matings. In these, *p* corresponds to the frequency of the 660-Kb deletion in the corresponding population. Assuming that the background rate of reproductive failure equals *f*, the extra rate of reproductive failure is expected to be (i) 0, (ii) 0, (iii) 0.25(1-*f*)*p* and (iv) 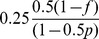, if all embryos that are homozygous for the 660-Kb deletion have died at the corresponding developmental stage. Figure 4 shows the observed versus expected extra rates of reproductive failure in the four mating types, assuming that *f* corresponds to the weighted average of the failure rate (at the corresponding days post-insemination) for mating types (i) and (ii), and *p* to the weighted average of the frequency of the deletion in the Nordic Red breeds included in the analysis. As expected, we observed a highly significant extra rate of reproductive failure ranging from ∼2% (p<10^−29^) at 35 days post-insemination to ∼5% (p<10^−154^) at 150 days post-insemination in mating type (iv). Comparing this extra rate with theoretical expectation computed as described above, indicates that 20% homozygous embryos have died before 35 days post-insemination and 79% before 150 days post-insemination. As we have demonstrated above that homozygosity for the 660 Kb deletion is fully lethal, this finding implies that a remaining 20% of homozygous embryos die between 150 days post-insemination and parturition. Mating type (iii) is expected to yield a proportion 0.25*p* of homozygous embryos as the sire is known to be carrier and the dam may have inherited the deletion from her ungenotyped mother (i.e. the maternal grand-dam). Performing the same comparison between observed and expected extra rate of reproductive failure in this mating type, yielded comparable estimates of embryonic death of homozygous embryos of 25% at 35 days and 88% at 150 days. In conclusion, increased rate of reproductive failure according to parental genotype supports the hypothesis that the 660Kb deletion is EL, causing fetal death between one and >5 months of gestation.

### The 660-Kb deletion is associated with a positive effect on milk yield and composition, which maintains it a high frequency in Nordic Red Cattle

**Figure 5:**
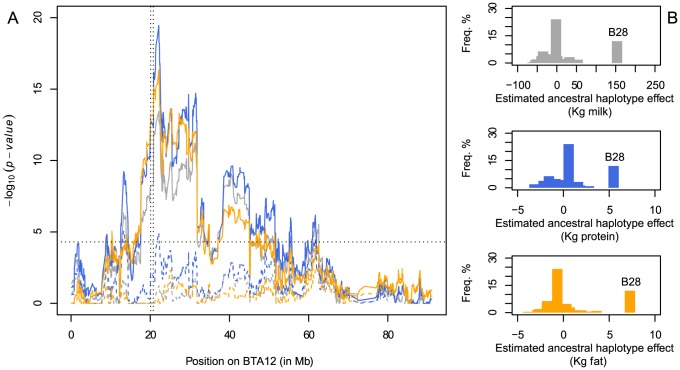
Detection of a QTL influencing milk production and composition on BTA12 in Nordic Red breeds. A. Haplotyped-based QTL mapping on BTA12 for milk (gray), protein (blue) and fat (orange) yield in Nordic Red cattle. The x-axis represents the physical position on BTA12 and the y-axis the p-value of the likelihood ratio test. Full and dashed lines represent QTL mapping with and without correction for haplotype B28 in strong association with the deletion. The vertical dotted lines indicate the position of the deletion whereas the horizontal line marks the chromosome-wide significance threshold. B. Effect and frequency of the 40 ancestral haplotypes on milk (gray), protein (blue) and fat (orange) yield (in Kg). Haplotype B28 tags the deletion.

The carrier frequencies observed in the three Nordic Red breeds (32%, 23% and 13%) are intriguingly high given the highly deleterious effect of the deletion. We reasoned that this might be due to a positive, direct or indirect effect of the deletion on desirable traits. We tested this hypothesis by scanning chromosome 12 for QTL influencing milk yield and composition using the same haplotype-based approach. We observed chromosome-wide significant QTL on milk, fat and protein yield in the three Nordic Red breeds (joint analysis), maximizing in the immediate vicinity of the 660-Kb deletion (Figure 5A). All QTLs were entirely driven by the strong positive effect of haplotype B28, previously shown to be associated with decreased fertility (Figure 5B). Indeed, including the B28 genotype as a fixed effect in the model annihilated the QTL effects on milk and fat yield, except for a small residual effect on protein (Figure 5A). Taken together, these findings suggest that the 660-Kb deletion is maintained at moderate to high frequency in Nordic Red breeds despite its deleterious effect on fertility because of its positive (direct or indirect) effect on milk yield and composition. The same pleiotropic effect on fertility and milk traits of a BTA12 QTL was previously reported in Norwegian Red [18].

## Discussion

We herein demonstrate that a QTL with major effect on fertility in Nordic Red cattle is due to the segregation of a 660-Kb deletion on chromosome 12 that is lethal in homozygous embryos. It demonstrates, somewhat counter-intuitively, that discernible Mendelian entities account for part of the inherited variation for this highly complex and lowly heritable trait.

Our work adds to the evidence that EL are at least in part responsible for the increase in insemination failure that is a growing concern in highly selected cattle populations. It is becoming apparent that, as human, domestic animals carry several deleterious alleles. In man, highly deleterious alleles are typically rare, and hence homozygosity for such variants is exceptional in the absence of consanguinity. In domestic animals, however, and as a result of intense selection and reduction in effective population sizes, a yet unidentified number of EL may be segregating at low to moderate frequencies in most populations. Assuming (i) that ∼25% of the increase in insemination failure is due to recessive EL and (ii) random mating, this could correspond for instance to 7.5 embryonic lethal equivalents segregating at 10% frequency, ∼30 embryonic lethal equivalents segregating at 5% frequency, or ∼750 embryonic lethal equivalents segregating at 1% frequency.

Fertility traits in livestock are typically modeled as being determined by (additive) breeding values of sire and dam. In fact, embryonic mortality caused by recessive EL alleles is determined by the non-additive genotype of the conceptus. The corresponding source of variation is only poorly captured by the additive parental breeding values, particularly when the population frequency of the corresponding EL is low. Accounting for the inbreeding coefficient of the conceptus may provide better estimates of the parental breeding values for fertility. It is noteworthy that the dam's fertility is often considered to be subject to inbreeding depression. This corresponds to the inbreeding coefficient of the dam, which is distinct from the inbreeding coefficient of the offspring.

In this work, we have used a traditional phenotype-driven forward genetic approach to map and subsequently dissect the QTL. Given the nature of the phenotype (difficult to observe embryonic lethality), a genotype-driven reverse genetic approach might be equally if not more appropriate. One way to achieve this is to search for significant, local depletions in autozygosity for haplotypes that are assumed to be associated with EL alleles. This approach has been successfully applied in the Holstein, Brown Swiss and Jersey breeds [13], [14]. It is noteworthy that several SNPs on both sides of the 660-Kb deletion were in significant Hardy-Weinberg disequilibrium as a result of excess heterozygosity (Figure S3). Moreover, there was a significant depletion in homozygosity for the B28 haplotype in Danish (p<1.616e-06), Swedish (p<1.570e-27) and Finnish (p<4.769e-52) Red cattle, indicating retrospectively that this approach could have been effective (particularly when combined with our Hidden Markov Model-based haplotyping method [19]) in detecting the corresponding deletion. However, to work this approach requires near complete LD (r^2^∼1) between the EL and the haplotype upon which it appears (which is unlikely always to be the case), as well as a very large study population (which is unlikely always to be available). We have recently proposed an alternative approach that might obviate some of these limitations [11]. In this approach, genome-wide NGS data obtained on a representative sample of moderate size of the population of interest are mined for predicted LoF variants. Candidate LoF variants, segregating at intermediate or even low frequency, are then genotyped on a much larger sample to test for Hardy-Weinberg disequilibrium (absence of homozygotes) and association with reduced fertility, two features expected for genuine EL variants. Until now attention has primarily focused on frame-shift, stop-gain, splice-site, and - to a lesser extent – highly disruptive missense variants. The present study indicates that the search for structural variants in NGS data, particularly deletions, might also be worth the effort.

In the specific case of the 660-Kb deletion uncovered in this work, we show that its high frequency is not only due to random drift, but also to the associated effect on milk yield and composition. This is at least the seventh example of balancing selection maintaining a deleterious allele at high frequency in livestock. Other examples include the *R615C* mutation in the porcine *RYR1* gene increasing muscle mass in heterozygotes yet causing the Porcine Stress and Pale Soft Exudative Meat Syndromes in homozygotes [23], *MSTN* LoF variants increasing muscle mass in heterozygotes yet causing dystocia in homozygotes [24], variants in *BMP15* and *GDF9* (members of the *TGFβ* family) increasing prolificity in heterozygous females yet causing infertility in homozygous ewes (e.g., [25], [26]), the *V700E* mutation in the ovine *FGFR3* gene increasing size in heterozygotes yet causing Spider Lamb in homozygotes [27]–[29], *MRC2* LoF variants increasing muscle mass in heterozygotes yet causing Crooked Tailed Syndrome in homozygotes [30], [31], and a LINE insertion in the porcine *SPEF2* gene that causes infertility in boars yet increases fertility in sows [32].

In all these examples, available evidence indicates that the same mutation and target gene underlie both the desirable effect in heterozygotes and undesirable effect in homozygotes. In the present case, we believe it most likely (because of its size) that the 660-Kb deletion also causes both embryonic lethality and favorable effect on milk yield and composition. However, we cannot exclude that the milk effect is due to a variant distinct from, yet in high linkage disequilibrium with the deletion. The residual effect on protein yield observed after correction for the deletion genotype tends to support the latter hypothesis.

Which target genes are responsible for the antagonistic effects on fertility and milk production remains to be determined. *RNASEH2B* is a strong candidate causative gene for the embryonic lethality as knocking it out causes embryonic death in the mouse [21], [22]. *RNASEH2B* (*ribonuclease H2, subunit B*) codes for the non catalytic subunit of RNase H2, an endonuclease that specifically degrades the RNA of RNA:DNA hybrids and participates in DNA replication. *RNASEH2B* loss-of-function mutations cause Aicardi-Goutieres syndrome type 2 in humans (AGS2, OMIM 610181). It remains possible, however, that one or the two other coding genes included in the deletion (*GUCY1B2* and *FAM124A*) or even *DLEU7* and the two non-coding RNA genes (*DLEU7-AS1*; *LINC00371*) contribute to the embryonic lethality as well. *GUCY1B2 (guanylate cyclase 1, soluble, beta 2)* codes for the widely expressed beta sub-unit of a nitric oxide-sensitive guanylyl cyclase of poorly defined function. Although apparently pseudogenized in humans, *GUCY1B2* is highly conserved in vertebrates including bovine. *FAM124A* codes for a protein conserved across vertebrates yet of unknown function. Human *DLEU7* is predicted to code for a “low quality protein” which, however, is poorly conserved in other mammals. Its function, as well as those of *DLEU7-AS1* and *LINC00371*, remain unknown. We can also not exclude the possibility that the deletion perturbs the expression of genes lying outside of it, and that this also affects embryonic development. Whether the effect on milk yield and composition is due to altered expression of one of the four genes in the deletion and/or one or more genes outside of the deletion remains unknown. It is worthwhile noting, however, that *FAM124A* is strongly expressed in myoepithelial cells of mammary gland [33].

Initial suspicion that a deletion might underlie the QTL came from the observation of significant deviation from Hardy-Weinberg equilibrium and inflation of parentage conflicts for a set of clustered SNPs. In most other studies that we are conducting, stringent quality control measures would have eliminated the corresponding markers prior to GWAS. While this would not have precluded the identification of the QTL, it would probably have hampered the discovery of the deletion. It thus seems advisable to at least verify whether SNPs that do not pass such stringent QC-tests are randomly scattered across the genome rather than clustered. The latter might be indicative of structural variants that deserve further analysis.

## Methods

### SNP genotyping

All animals were genotyped using the BovineSNP50 beadchip (Illumina, San Diego, CA), which assayed 54,001 SNP markers, at Aarhus University and GenoScan A/S, Denmark. Genomic DNA was extracted from whole blood or semen. The Illumina Infinium II multi-sample assay protocol was followed to prepare SNP chips for scanning using the iScan imaging system. Analysis was performed using Beadstudio software (version 3.1). SNP positions within a chromosome were designated according to the *Bos taurus* genome UMD3.1 assembly [34]. The quality parameters used for selection of SNPs in the study were minimum call rates of 90% for individuals and 95% for loci. Marker loci with minor allele frequencies (MAFs) below 5% were excluded for SNP-by-SNP association analysis. The minimum acceptable Beadstudio Gencall (GC) score (see http://res.illumina.com/documents/products/technotes/technote_infinium_genotyping_data_analysis.pdf for more details) was 0.60 for individual typing, and individuals with average GC scores below 0.65 were excluded. After quality control the whole genome map reduced to 37,123 common SNPs across the five breeds analyzed.

For the breed-wise analysis on BTA12, 1166 SNPs passed the filtering for call rates. After filtering for MAF >0.05 there were 1095, 983, 1125, 1107, 1093 SNPs for Holstein, Jersey, Danish Red, Swedish Red and Finnish Red cattle respectively. In the second phasing stage, 17 markers presenting more than 10 parentage conflicts were removed from the analysis.

In addition, 243 Finnish Red bulls were genotyped on the Bovine HD Genotyping BeadChip (Illumina, San Diego, CA) with 725,293 SNPs mapping on autosomes. SNP positions were designated according to the *Bos taurus* genome UMD 3.1.

### Phenotypes

We used phenotypic data from three Nordic cattle breeds (Holstein, Jersey and Nordic Red). Fertility traits analyzed in this study and number of individuals with records are described in Text S2. For details regarding the phenotypes recorded and models used in routine breeding value prediction, see http://www.nordicebv.info.

### Across-breed genome-wide association scan

A whole genome scan was performed to test for the presence of fertility QTL in a multi-breed data set comprised of Holstein, Jersey, Red dairy cattle of Denmark, Sweden and Finland. The effect of each SNP was estimated by successively fitting the following linear mixed model
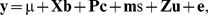
where **y** is the vector of estimated breeding values (EBVs) of the bulls for the fertility index, μ is the overall mean, **b** is the vector of breed effects, **P** is the matrix of the four top principal components (estimated as in [35] from the genome wide markers), **c** is the vector of effects of the principal components, **m** is the vector of additively coded SNP genotypes, s is the allele substitution effect of the SNP, **u** is the vector of random sire effects assumed to be 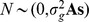, where 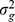 is the additive genetic variance and **A_s_** is the additive genetic relationship among the sires of the bulls derived from the pedigree, and **e** is the vector of random individual error term assumed to be 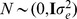.

The allele substitution effect s was estimated by AI-REML implemented in DMU [36] and its significance was estimated using a t-test. The genome-wide significance threshold corresponding to a familywise error rate of 0.05, was set at p<1e-6 after correction for multiple testing using a Bonferroni correction for 50,000 independent tests.

### Chromosome-specific haplotype-based association analysis

Genotypes were first phased and clustered into ancestral haplotypes with the PHASEBOOK software package [19]. The SNPs were first phased utilizing information from pedigree with LINKPHASE [19], and then using LD with DAGPHASE [19] and Beagle [37]. The phased genotypes were then clustered into 40 ancestral haplotypes using HIDDENPHASE [19].

A haplotype-based association was then carried out using these ancestral haplotypes at each SNP position on BTA12. The presence of a QTL was tested using the following linear mixed model.
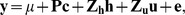
Where **y** are the de-regressed proofs (e.g., [38]) for the fertility traits, μ is the overall mean, **P** is the matrix of the four top principal components estimated from the genome wide markers, **c** is the vector of effects of principal components, **h** is a vector of 40 random ancestral haplotype effects with variance 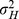 assumed to be 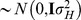, **I** is an identity matrix, **u** is the vector of individual polygenic effects with variance 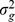 assumed to be 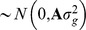, **A** is the additive relationship matrix estimated from the pedigree, and **e** is the vector of individual error terms with variance 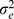 assumed to be 
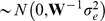
, **W** is the diagonal matrix containing weights derived from the reliabilities (r^2^) of the de-regressed EBVs (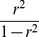) . The variances 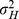, 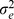 and 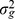 were estimated using AI-REML implemented in DMU software package [36], and the presence of a QTL was tested using a Likelihood Ratio Test (distributed as a chi-square distribution with 1 df). The chromosome-wide significance threshold corresponding to a familywise error rate of 0.05 was set at p<0.00005 after correction for multiple testing using a Bonferroni correction for 1,000 independent tests.

### Analysis of the rate of insemination failure as a function of the genotypes of sires and maternal grand-sires for the deletion

A linear mixed model was applied to test the effect of mating type on the rate of reproductive failure established by the fact that the cows returns in oestrus at 35, 56, 100 and 150 days after insemination. Four classes of matings were defined according to 660-kb deletion genotype: (i) non-carrier (NC) sire X daughter of NC maternal grand-sire, (ii) non-carrier (NC) sire X daughter of carrier (C) maternal grand-sire, (iii) C sire X daughter of NC maternal grand-sire, and (iv) C sire X daughter of C maternal grand-sire. Only genotyped bulls for which the genotype of the 660-kb deletion could be predicted based on the haplotype B28 were used. A total of 3,157,753 inseminations were analyzed (1,936,585, 590,806, 443,464 and 186,898 for mating types i, ii, iii and iv, respectively). The average rate of insemination failure was 0.278, 0.396, 0.475 and 0.493 at, respectively, 35, 56, 100 and 150 days after insemination.

The fitted mixed model included parity and month of insemination (by year) as fixed effect, and maternal grand-sire as random effect:
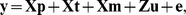
where **y** is a vector indicating return to oestrus 35, 56, 100 or 150 days after insemination (0 in case of success and 1 in case of failure), **p** is the vector of effects of parity, **t** is the vector of effects of month and year of insemination, **m** is the vector of effects of mating type, **u** is the random sire effect assumed to be 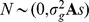; where 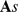 is the additive genetic relationship among the sires of the dams derived from the pedigree, and **e** is the vector of random individual error terms assumed to be 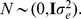


In a population where the deletion has a frequency *p*, the proportion of carriers is 2*p*. In mating types (i) and (ii) the probability that both parents are carriers is null (since the sires are non-carriers). In class (iii) and (iv) the maternal grand-dam has probability 2*p* to be carrier. As a result in class (iii) the dam has a probability *p* to be carrier. Finally, in class (iv) the maternal grand-sire is a carrier and has 0.5 chance to transmit the deletion whereas the maternal grand-dam has a probability *p* to transmit the deletion to the dam. As a consequence, the dam has probability 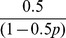 to be carrier (since dams cannot be homozygotes for the deletion). The expected proportion of conceptuses that are homozygous for the 660-Kb deletion is equal to 0.25 multiplied by the probability that both parents are carriers, corresponding respectively to (i) 0, (ii) 0, (iii) 0.25*p*, and (iv) 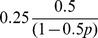 for the four different mating types.

### Next generation sequencing - Danish Red samples

DNA samples were extracted at Aarhus University (Foulum) from semen samples using standard procedures. Sequencing was done using Illumina sequencers at Beijing Genomics Institute (Shenzhen, China). Sequencing was shotgun paired-end sequencing with a read length of 91 base pairs. Fastq data were converted from Illumina to Sanger quality encoding using a patched version of MAQ [39]. They were aligned to the UMD3.1 assembly of the cattle genome [34] using BWA [40] version 0.6.2. They were converted to raw BAM files using samtools [41]. Quality scores were re-calibrated using the Genome Analysis Toolkit version 1.6 [42] following the Human 1000 Genome guidelines incorporating information from dbSNP version 133 [43]. Sequences were realigned around insertion/deletions using the Genome Analysis Toolkit version 1.6. Variants were called using the Genome Analysis Toolkit version 1.6.

### Next generation sequencing - Finnish Ayrshire samples

Genomic DNA from 18 Finnish Ayrshire bulls was extracted and purified according to standard protocols. Sample preparation, cluster generation and sequencing were performed according to the manufacturer's protocols (Illumina Paired-End Cluster Generation kit (version 4)). Briefly, two paired-end libraries were prepared and sequenced on a HiSeq2000 (Illumina, San Diego, California, USA). Genomic DNA was sheared by nebulization, ligated with Illumina's PE adaptors, and fragments approximately 300 and 800 bases in length were gel purified followed by PCR amplification and column purification. Purity and yield were checked using a 2100 Bioanalyzer (Agilent Technologies, Santa Clara, California, USA) and yields were additionally measured using a Qubit (Invitrogen, Carlsbad, California, USA). Fastq files were generated using the Illumina data analysis workflow software Casava versions 1.7 and 1.8, and base qualities of a subset of reads from each sequencing lane were visually inspected using FastQC (http://www.bioinformatics.babraham.ac.uk/projects/fastqc/). Each bull was sequenced to an approximate coverage of 20×. We used the UMD3.1 assembly of the cow genome as a reference sequence for mapping (http://stothard.afns.ualberta.ca/1000_bull_genomes/reference_for_mapping/umd_3_1_reference_1000_bull_genomes.fa.gz). Sequencing reads were aligned to the cow reference genome using BWA (0.5.9-r16; [40]) with default parameters. Merging of BAM files and duplicate filtering was performed using Picard (version 1.67; http://picard.sourceforge.net). Filtering for mapping quality was done during variant detection with the Genome Analysis Toolkit version 2 (http://www.broadinstitute.org/gatk/; [42]) including indel realignment and base score recalibration.

### Breakpoint amplification and sequencing

Genomic DNA was extracted from frozen sperm straws of two carrier and two homozygous wild-type sires using the MagAttract Mini M48 Kit (Qiagen). PCR amplification was carried out with the Phusion Hi-Fidelity PCR Kit (New England BioLabs, Ipswich) in a 20 µl volume of 1× Phusion buffer, 3% of DMSO, 0.5 mM dNTP, 0.5 µM primer mix (forward primer: 5′-CGA ATT CTA TTT CTG AAA GGG GAA A-3′ and reverse primer: 5′-TTT GTC TTA CAT ATT GCG GCA CTC-3′) and 20 ng of genomic DNA. The cycling conditions were the following: (i) an initial denaturation of 98°C for 30 sec, (ii) 10 cycles of 10 sec denaturation (98°C), 30 sec hybridization (70°C with 1°C decrease at each cycle), 30 sec elongation (72°C), (iii) 25 cycles of 10 sec denaturation (98°C), 30 sec hybridization (60°C), 30 sec elongation (72°C) and a final 7 min elongation (72°C). PCR products were separated a on a 1.5% agarose gel, purified and directly sequenced using the Big Dye terminator cycle sequencing kit (Applied Biosystems, Foster City, CA). Electrophoresis of sequencing reactions was performed on an ABI PRISM 3730 DNA analyzer (PE Applied Biosystems, Foster City, CA). Sequence traces were visualized using the CodonCode Aligner 4.1 software (LI-COR, Inc.). A 318 bp control amplification, with a primer pair within the deletion (forward primer: 5′-AGC TGC TTC TCG GAA GGG AC-3′ and reverse primer: 5′-CAG GAG TAC GCT ACT AAC AC-3′), was performed in parallel using standard PCR conditions.

## Supporting Information

Figure S1 Fine-mapping of the fertility QTL on BTA12. Haplotyped-based QTL fine-mapping on BTA12 in Finnish Ayrshire (red) and Swedish Red (gray) cattle. The x-axis represents the physical position on BTA12 and the y-axis the p-value of the likelihood ratio test. The horizontal line represents the genome-wide significance threshold. Histograms on the right describe the effect (x-axis) and frequency (y-axis) of the 40 modeled ancestral haplotypes in Finnish Ayrshire (red) and Swedish Red (gray) cattle. Haplotype A27 is associated with the deletion. A: Number of inseminations in cows (AISC), B: Number of inseminations in heifers (AISH), C: Interval from calving to first insemination (ICF), D: Interval between first and last insemination in cows (IFLC), E: Interval between first and last insemination in heifers (IFLH), F: Non-return rate at 56 days in cows (NRRC) and G: Non-return rate at 56 days in heifers (NRRH).
(TIFF)

Figure S2 Comparison of haplotypes A27 and B28 around the 660-Kb deletion. The allele-specific emission probabilities of ancestral haplotypes A27 and B28 are shown for 150 markers spanning positions 17,121,591 to 28,647,560. Each base is represented by a different color and the size of the letter is function of its emission probability. Major differences between ancestral haplotypes A27 and B28 are marked by red arrows. For ancestral haplotype B28, missing positions correspond to markers that have been discarded as a result of more stringent QC prior to the second analysis. The position of the 660 Kb deletion is shown by a red box. We can observe that 1) for most positions, the allele carried by both haplotypes are well defined, 2) for a window of almost 10 Mb encompassing the deletion, ancestral haplotypes A27 and B28 are identical (except in the deletion) and 3) within the deletion, haplotype A27 is poorly defined since the observed homozygous alleles corresponded to the homologous chromosome (haplotype A27 carries a null allele and genotypes are incorrectly called homozygous). The stretch of five markers in the deletion causes difficulties in assigning haplotypes to the correct ancestral haplotypes and removing these markers allows more accurate assignment of haplotypes to ancestral haplotypes. Note that the method does not require an individual carrying the deletion to have a haplotype identical to ancestral haplotype B28 over the entire chromosome to have it assigned locally to B28.
(TIF)

Figure S3 First evidence suggesting a deletion based on genotyping data. A. Deviation from Hardy-Weinberg equilibrium for SNPs on BTA12. Red (green) dots represent excess of homozygosity (heterozygosity). B. Number of identified parentage conflicts (Mendelian inconsistencies between parent and offspring) per marker on BTA12.
(EPS)

Figure S4 Distribution of distance separating paired-end reads for the sequenced Danish Red and Finnish Ayrshire sires. Two different fragment lengths were purified and amplified during library preparation (see Material and Methods).
(TIF)

Figure S5 A. Proximal and distal breakpoint boundaries supported by paired-reads bridging the deletion and split reads displayed as zoomed-in IGV screen captures: (a) coverage depth of a carrier animal in a 2 Mb region containing the deletion; (b) zoom of the proximal and distal BP regions for the same animal with paired-reads bridging the deletion highlighted in dark brown and, below, a track showing the repeat context (blue lines) of the proximal and distal BP respectively lying within a 735 bp L1ME1 and a 5253 bp L1BT elements; (c) zoom of the proximal and distal BP regions showing split reads marked by an asterisk. B. PCR amplification across (left panel) and within the deletion (right panel) for two homozygous wild-type (+/+), two carriers (Δ/+) animals. neg: negative control (neg). M: molecular weight marker (Smart Ladder, Eurogentec Inc.).
(TIF)

Figure S6 Fine-mapping of the fertility QTL on BTA12. Haplotyped-based QTL fine-mapping on BTA12 in Finnish Ayrshire (red) and Swedish Red (gray) cattle. The x-axis represents the physical position on BTA12 and the y-axis the p-value of the likelihood ratio test. Full and dashed lines represent QTL mapping with and without correction for haplotype B28. The vertical dotted lines indicate the position of the deletion. The horizontal line represents the chromosome-wide significance threshold. The histograms describe the effect and frequency of the 40 ancestral haplotypes in Finnish Ayrshire (red) and Swedish Red (gray) cattle. The haplotype B28 is carrier of the deletion: A. Number of inseminations (cows), B. Number of inseminations (heifers), C. Interval from calving to first insemination, D. Interval between first and last insemination (heifers), E. Non-return rate at 56 days (cows) and F. Non-return rate at 56 days (heifers).
(TIFF)

Text S1 Deletion genotype calling based on total signal intensity, homozygosity, parentage conflicts and haplotype. This document explains how we exploited signal intensity, obligate homozygosity and parentage conflicts for the markers in the deletion to confidently genotype 2,139 Finnish Ayrshire, 1,221 Swedish Red and 1,096 Danish Red sires for the deletion. In addition, it describes estimation of linkage disequilibrium (r^2^) between the deletion and haplotypes B28 or A27 and estimation of the frequency of the deletion in different Nordic Red cattle populations.
(PDF)

Text S2 Description of fertility traits. This document gives a brief description of all fertility traits used in the present study (including the number of genotyped individuals with records per trait).
(PDF)

## References

[1] Dekkers JC, Hospital F (2002) The use of molecular genetics in the improvement of agricultural populations. Nat Rev Genet 3: 22–32. 11823788 10.1038/nrg701

[2] Grisart B, Coppieters W, Farnir F, Karim L, Ford C, et al (2002) Positional candidate cloning of a QTL in dairy cattle: identification of a missense mutation in the bovine DGAT1 gene with major effect on milk yield and composition. Genome Res 12: 222–231. 11827942 10.1101/gr.224202

[3] Hayes BJ, Pryce J, Chamberlain AJ, Bowman PJ, Goddard ME (2010) Genetic architecture of complex traits and accuracy of genomic prediction: coat colour, milk-fat percentage, and type in Holstein cattle as contrasting model traits. PLoS Genet 6: e1001139. 20927186 10.1371/journal.pgen.1001139 PMC2944788

[4] Kemper KE, Goddard ME (2012) Understanding and predicting complex traits: knowledge from cattle. Hum Mol Genet 21: R45–51. 22899652 10.1093/hmg/dds332

[5] Washburn SP, Silvia WJ, Brown CH, McDaniel BT, McAllister AJ (2002) Trends in reproductive performance in Southeastern Holstein and Jersey DHI herds. J Dairy Sci 85: 244–251. 11860117 10.3168/jds.S0022-0302(02)74073-3

[6] Lucy MC (2001) Reproductive loss in high-producing dairy cattle: where will it end? J Dairy Sci 84: 1277–1293. 11417685 10.3168/jds.S0022-0302(01)70158-0

[7] Silvia W (1998) Changes in reproductive performance of Holstein dairy cows in Kentucky from 1972 to 1996. J Dairy Sci 81 (Suppl. 1) 244.

[8] Sun C, Madsen P, Lund MS, Zhang Y, Nielsen US, et al (2010) Improvement in genetic evaluation of female fertility in dairy cattle using multiple-trait models including milk production traits. J Anim Sci 88: 871–878. 19966172 10.2527/jas.2009-1912

[9] MacArthur DG, Balasubramanian S, Frankish A, Huang N, Morris J, et al (2012) A systematic survey of loss-of-function variants in human protein-coding genes. Science 335: 823–828. 22344438 10.1126/science.1215040 PMC3299548

[10] Bittles AH, Neel JV (1994) The costs of human inbreeding and their implications for variations at the DNA level. Nat Genet 8: 117–121. 7842008 10.1038/ng1094-117

[11] Charlier C, Agerholm JS, Coppieters W, Karlskov-Mortensen P, Li W, et al (2012) A deletion in the bovine FANCI gene compromises fertility by causing fetal death and brachyspina. PLoS One 7: e43085. 22952632 10.1371/journal.pone.0043085 PMC3430679

[12] Thomsen B, Horn P, Panitz F, Bendixen E, Petersen AH, et al (2006) A missense mutation in the bovine SLC35A3 gene, encoding a UDP-N-acetylglucosamine transporter, causes complex vertebral malformation. Genome Res 16: 97–105. 16344554 10.1101/gr.3690506 PMC1356133

[13] VanRaden PM, Olson KM, Null DJ, Hutchison JL (2011) Harmful recessive effects on fertility detected by absence of homozygous haplotypes. J Dairy Sci 94: 6153–6161. 22118103 10.3168/jds.2011-4624

[14] Sonstegard TS, Cole JB, VanRaden PM, Van Tassell CP, Null DJ, et al (2013) Identification of a nonsense mutation in CWC15 associated with decreased reproductive efficiency in Jersey cattle. PLoS One 8: e54872. 23349982 10.1371/journal.pone.0054872 PMC3551820

[15] Fritz S, Capitan A, Djari A, Rodriguez SC, Barbat A, et al (2013) Detection of Haplotypes Associated with Prenatal Death in Dairy Cattle and Identification of Deleterious Mutations in GART, SHBG and SLC37A2. PLoS One 8: e65550. 23762392 10.1371/journal.pone.0065550 PMC3676330

[16] Schulman NF, Sahana G, Lund MS, Viitala SM, Vilkki JH (2008) Quantitative trait loci for fertility traits in Finnish Ayrshire cattle. Genet Sel Evol 40: 195–214. 18298935 10.1186/1297-9686-40-2-195 PMC2674925

[17] Schulman NF, Sahana G, Iso-Touru T, McKay SD, Schnabel RD, et al (2011) Mapping of fertility traits in Finnish Ayrshire by genome-wide association analysis. Anim Genet 42: 263–269. 21554346 10.1111/j.1365-2052.2010.02149.x

[18] Olsen HG, Hayes BJ, Kent MP, Nome T, Svendsen M, et al (2011) Genome-wide association mapping in Norwegian Red cattle identifies quantitative trait loci for fertility and milk production on BTA12. Anim Genet 42: 466–474. 21906098 10.1111/j.1365-2052.2011.02179.x

[19] Druet T, Georges M (2010) A hidden markov model combining linkage and linkage disequilibrium information for haplotype reconstruction and quantitative trait locus fine mapping. Genetics 184: 789–798. 20008575 10.1534/genetics.109.108431 PMC2845346

[20] Hou Y, Bickhart DM, Hvinden ML, Li C, Song J, et al (2012) Fine mapping of copy number variations on two cattle genome assemblies using high density SNP array. BMC Genomics 13: 376. 22866901 10.1186/1471-2164-13-376 PMC3583728

[21] Reijns MA, Rabe B, Rigby RE, Mill P, Astell KR, et al (2012) Enzymatic removal of ribonucleotides from DNA is essential for mammalian genome integrity and development. Cell 149: 1008–1022. 22579044 10.1016/j.cell.2012.04.011 PMC3383994

[22] White JK, Gerdin AK, Karp NA, Ryder E, Buljan M, et al (2013) Genome-wide Generation and Systematic Phenotyping of Knockout Mice Reveals New Roles for Many Genes. Cell 154: 452–464. 23870131 10.1016/j.cell.2013.06.022 PMC3717207

[23] Fujii J, Otsu K, Zorzato F, de Leon S, Khanna VK, et al (1991) Identification of a mutation in porcine ryanodine receptor associated with malignant hyperthermia. Science 253: 448–451. 1862346 10.1126/science.1862346

[24] Georges M (2012) Impact of high-throughput genotyping and sequencing on the identification of genes and variants underlying phenotypic variation in domestic cattle. In: Womack J, editor. Bovine Genomics. Oxford (UK): Wiley-Blackwell.

[25] Galloway SM, McNatty KP, Cambridge LM, Laitinen MP, Juengel JL, et al (2000) Mutations in an oocyte-derived growth factor gene (BMP15) cause increased ovulation rate and infertility in a dosage-sensitive manner. Nat Genet 25: 279–283. 10888873 10.1038/77033

[26] Hanrahan JP, Gregan SM, Mulsant P, Mullen M, Davis GH, et al (2004) Mutations in the genes for oocyte-derived growth factors GDF9 and BMP15 are associated with both increased ovulation rate and sterility in Cambridge and Belclare sheep (Ovis aries). Biol Reprod 70: 900–909. 14627550 10.1095/biolreprod.103.023093

[27] Cockett NE, Shay TL, Beever JE, Nielsen D, Albretsen J, et al (1999) Localization of the locus causing Spider Lamb Syndrome to the distal end of ovine Chromosome 6. Mamm Genome 10: 35–38. 9892730 10.1007/s003359900938

[28] Beever JE, Smit MA, Meyers SN, Hadfield TS, Bottema C, et al (2006) A single-base change in the tyrosine kinase II domain of ovine FGFR3 causes hereditary chondrodysplasia in sheep. Anim Genet 37: 66–71. 16441300 10.1111/j.1365-2052.2005.01398.x

[29] Smith LB, Dally MR, Sainz RD, Rodrigue KL, Oberbauer AM (2006) Enhanced skeletal growth of sheep heterozygous for an inactivated fibroblast growth factor receptor 3. J Anim Sci 84: 2942–2949. 17032787 10.2527/jas.2006-255

[30] Fasquelle C, Sartelet A, Li W, Dive M, Tamma N, et al (2009) Balancing selection of a frame-shift mutation in the MRC2 gene accounts for the outbreak of the Crooked Tail Syndrome in Belgian Blue Cattle. PLoS Genet 5: e1000666. 19779552 10.1371/journal.pgen.1000666 PMC2739430

[31] Sartelet A, Klingbeil P, Franklin CK, Fasquelle C, Geron S, et al (2012) Allelic heterogeneity of Crooked Tail Syndrome: result of balancing selection? Anim Genet 43: 604–607. 22497452 10.1111/j.1365-2052.2011.02311.x

[32] Sironen A, Uimari P, Iso-Touru T, Vilkki J (2012) L1 insertion within SPEF2 gene is associated with increased litter size in the Finnish Yorkshire population. J Anim Breed Genet 129: 92–97. 22394230 10.1111/j.1439-0388.2011.00977.x

[33] Uhlen M, Oksvold P, Fagerberg L, Lundberg E, Jonasson K, et al (2010) Towards a knowledge-based Human Protein Atlas. Nat Biotechnol 28: 1248–1250. 21139605 10.1038/nbt1210-1248

[34] Zimin AV, Delcher AL, Florea L, Kelley DR, Schatz MC, et al (2009) A whole-genome assembly of the domestic cow, Bos taurus. Genome Biol 10: R42. 19393038 10.1186/gb-2009-10-4-r42 PMC2688933

[35] Price AL, Patterson NJ, Plenge RM, Weinblatt ME, Shadick NA, et al (2006) Principal components analysis corrects for stratification in genome-wide association studies. Nat Genet 38: 904–909. 16862161 10.1038/ng1847

[36] Madsen P, Jensen J (2010) DMU, A package for analysing Multivariate Mixed Models. http://dmuagrscidk/dmuv6_guide50pdf Version 6, release 5.0.

[37] Browning BL, Yu Z (2009) Simultaneous genotype calling and haplotype phasing improves genotype accuracy and reduces false-positive associations for genome-wide association studies. Am J Hum Genet 85: 847–861. 19931040 10.1016/j.ajhg.2009.11.004 PMC2790566

[38] Andersson-Eklund L, Danell B (1993) Associations of breeding values for disease traits and genetic markers in dairy cattle estimated with a mixed model. J Dairy Sci 76: 3785–3791. 8132886 10.3168/jds.S0022-0302(93)77722-X

[39] Li H, Ruan J, Durbin R (2008) Mapping short DNA sequencing reads and calling variants using mapping quality scores. Genome Res 18: 1851–1858. 18714091 10.1101/gr.078212.108 PMC2577856

[40] Li H, Durbin R (2009) Fast and accurate short read alignment with Burrows-Wheeler transform. Bioinformatics 25: 1754–1760. 19451168 10.1093/bioinformatics/btp324 PMC2705234

[41] Li H, Handsaker B, Wysoker A, Fennell T, Ruan J, et al (2009) The Sequence Alignment/Map format and SAMtools. Bioinformatics 25: 2078–2079. 19505943 10.1093/bioinformatics/btp352 PMC2723002

[42] McKenna A, Hanna M, Banks E, Sivachenko A, Cibulskis K, et al (2010) The Genome Analysis Toolkit: a MapReduce framework for analyzing next-generation DNA sequencing data. Genome Res 20: 1297–1303. 20644199 10.1101/gr.107524.110 PMC2928508

[43] Sherry ST, Ward M, Sirotkin K (2000) Use of molecular variation in the NCBI dbSNP database. Hum Mutat 15: 68–75. 10612825 10.1002/(SICI)1098-1004(200001)15:1<68::AID-HUMU14>3.0.CO;2-6

[44] Trapnell C, Roberts A, Goff L, Pertea G, Kim D, et al (2012) Differential gene and transcript expression analysis of RNA-seq experiments with TopHat and Cufflinks. Nat Protoc 7: 562–578. 22383036 10.1038/nprot.2012.016 PMC3334321

