# Supplementary figures and images for: A 660-Kb Deletion with Antagonistic Effects on Fertility and Milk Production Segregates at High Frequency in Nordic Red Cattle: Additional Evidence for the Common Occurrence of Balancing Selection in Livestock
Source: PLoS Genet. 2014 Jan 2;10(1):e1004049. doi: 10.1371/journal.pgen.1004049 (PMC3879169; doi:10.1371/journal.pgen.1004049)

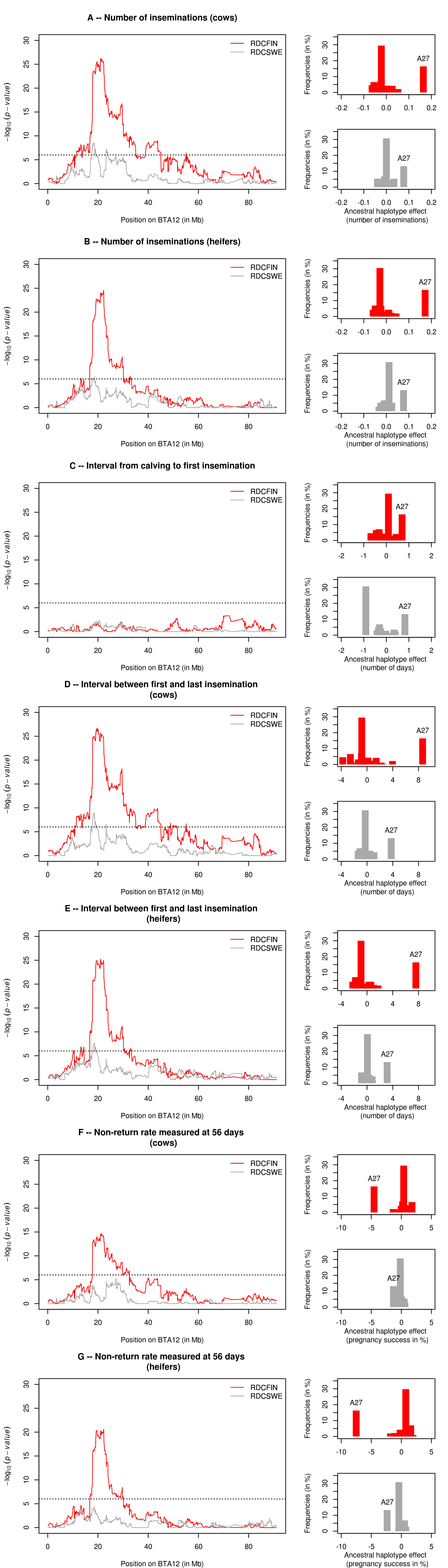

Supplement: Figure S1 — Fine-mapping of the fertility QTL on BTA12. Haplotyped-based QTL fine-mapping on BTA12 in Finnish Ayrshire (red) and Swedish Red (gray) cattle. The x-axis represents the physical position on BTA12 and the y-axis the p-value of the likelihood ratio test. The horizontal line represents the genome-wide significance threshold. Histograms on the right describe the effect (x-axis) and frequency (y-axis) of the 40 modeled ancestral haplotypes in Finnish Ayrshire (red) and Swedish Red (gray) cattle. Haplotype A27 is associated with the deletion. A: Number of inseminations in cows (AISC), B: Number of inseminations in heifers (AISH), C: Interval from calving to first insemination (ICF), D: Interval between first and last insemination in cows (IFLC), E: Interval between first and last insemination in heifers (IFLH), F: Non-return rate at 56 days in cows (NRRC) and G: Non-return rate at 56 days in heifers (NRRH). (TIFF) [file pgen.1004049.s001.tiff]

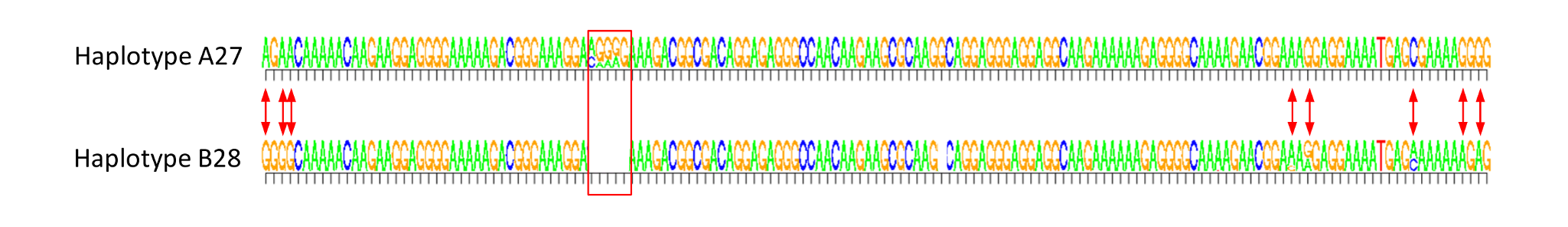

Supplement: Figure S2 — Comparison of haplotypes A27 and B28 around the 660-Kb deletion. The allele-specific emission probabilities of ancestral haplotypes A27 and B28 are shown for 150 markers spanning positions 17,121,591 to 28,647,560. Each base is represented by a different color and the size of the letter is function of its emission probability. Major differences between ancestral haplotypes A27 and B28 are marked by red arrows. For ancestral haplotype B28, missing positions correspond to markers that have been discarded as a result of more stringent QC prior to the second analysis. The position of the 660 Kb deletion is shown by a red box. We can observe that 1) for most positions, the allele carried by both haplotypes are well defined, 2) for a window of almost 10 Mb encompassing the deletion, ancestral haplotypes A27 and B28 are identical (except in the deletion) and 3) within the deletion, haplotype A27 is poorly defined since the observed homozygous alleles corresponded to the homologous chromosome (haplotype A27 carries a null allele and genotypes are incorrectly called homozygous). The stretch of five markers in the deletion causes difficulties in assigning haplotypes to the correct ancestral haplotypes and removing these markers allows more accurate assignment of haplotypes to ancestral haplotypes. Note that the method does not require an individual carrying the deletion to have a haplotype identical to ancestral haplotype B28 over the entire chromosome to have it assigned locally to B28. (TIF) [file pgen.1004049.s002.tiff]

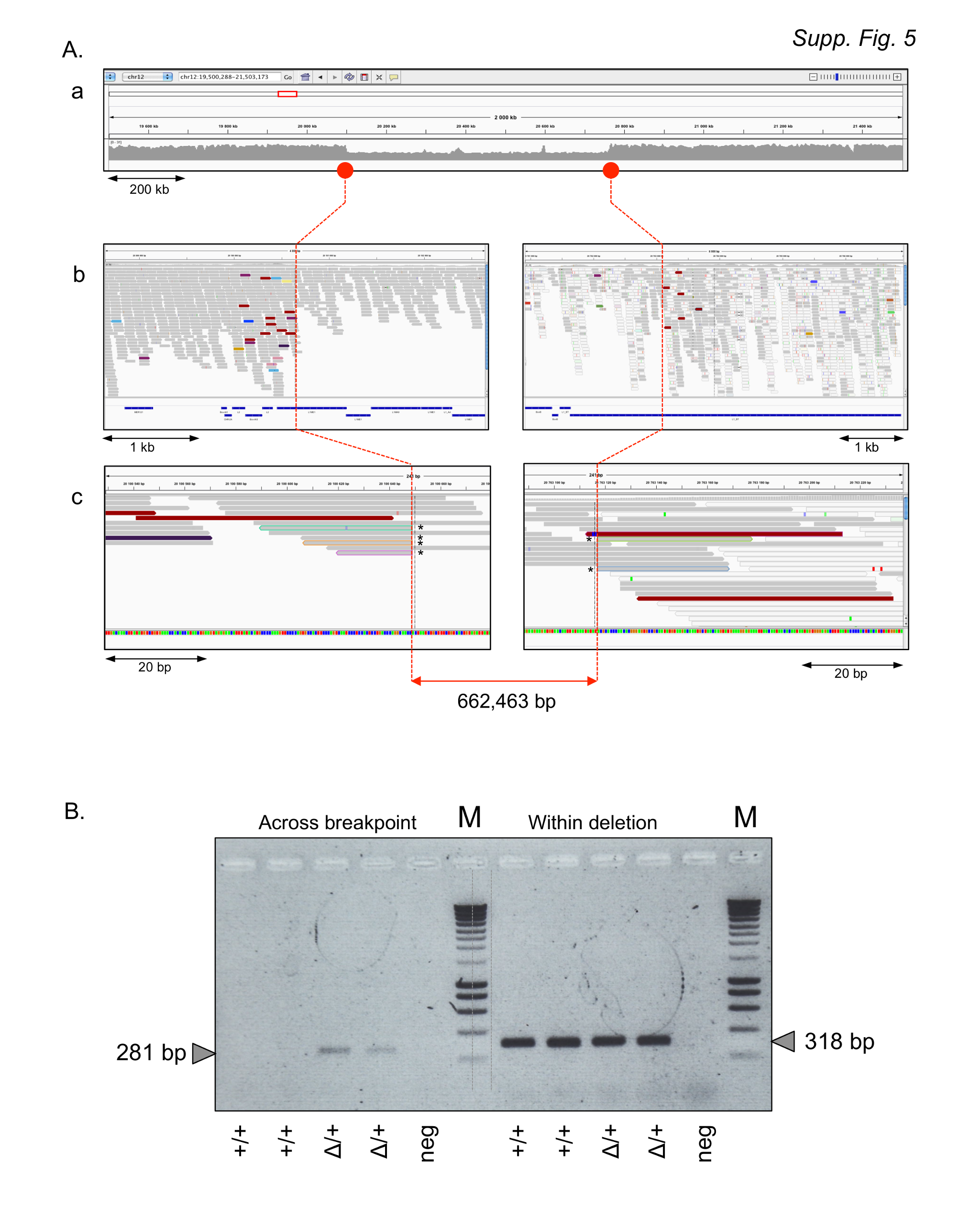

Supplement: Figure S5 — A. Proximal and distal breakpoint boundaries supported by paired-reads bridging the deletion and split reads displayed as zoomed-in IGV screen captures: (a) coverage depth of a carrier animal in a 2 Mb region containing the deletion; (b) zoom of the proximal and distal BP regions for the same animal with paired-reads bridging the deletion highlighted in dark brown and, below, a track showing the repeat context (blue lines) of the proximal and distal BP respectively lying within a 735 bp L1ME1 and a 5253 bp L1BT elements; (c) zoom of the proximal and distal BP regions showing split reads marked by an asterisk. B. PCR amplification across (left panel) and within the deletion (right panel) for two homozygous wild-type (+/+), two carriers (Δ/+) animals. neg: negative control (neg). M: molecular weight marker (Smart Ladder, Eurogentec Inc.). (TIF) [file pgen.1004049.s005.tif]

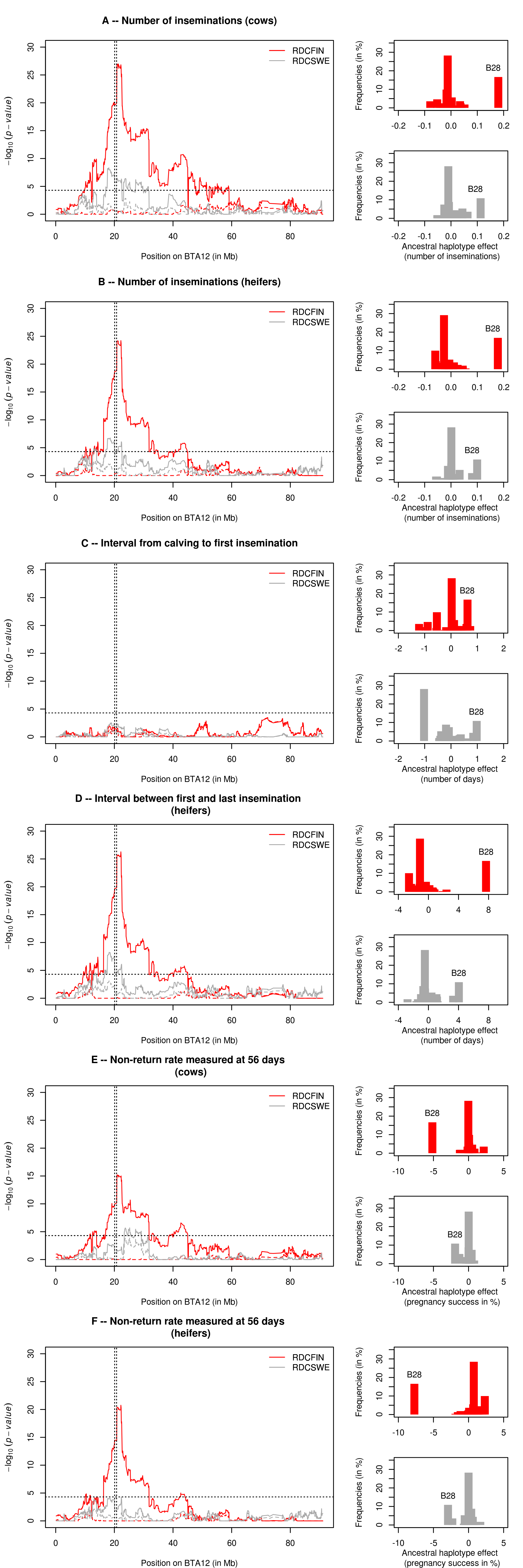

Supplement: Figure S6 — Fine-mapping of the fertility QTL on BTA12. Haplotyped-based QTL fine-mapping on BTA12 in Finnish Ayrshire (red) and Swedish Red (gray) cattle. The x-axis represents the physical position on BTA12 and the y-axis the p-value of the likelihood ratio test. Full and dashed lines represent QTL mapping with and without correction for haplotype B28. The vertical dotted lines indicate the position of the deletion. The horizontal line represents the chromosome-wide significance threshold. The histograms describe the effect and frequency of the 40 ancestral haplotypes in Finnish Ayrshire (red) and Swedish Red (gray) cattle. The haplotype B28 is carrier of the deletion: A. Number of inseminations (cows), B. Number of inseminations (heifers), C. Interval from calving to first insemination, D. Interval between first and last insemination (heifers), E. Non-return rate at 56 days (cows) and F. Non-return rate at 56 days (heifers). (TIFF) [file pgen.1004049.s006.tiff]
