# Supplementary material for: A 660-Kb Deletion with Antagonistic Effects on Fertility and Milk Production Segregates at High Frequency in Nordic Red Cattle: Additional Evidence for the Common Occurrence of Balancing Selection in Livestock
Source: PLoS Genet. 2014 Jan 2;10(1):e1004049. doi: 10.1371/journal.pgen.1004049 (PMC3879169; doi:10.1371/journal.pgen.1004049)
Supplement: Text S1 — Deletion genotype calling based on total signal intensity, homozygosity, parentage conflicts and haplotype. This document explains how we exploited signal intensity, obligate homozygosity and parentage conflicts for the markers in the deletion to confidently genotype 2,139 Finnish Ayrshire, 1,221 Swedish Red and 1,096 Danish Red sires for the deletion. In addition, it describes estimation of linkage disequilibrium (r2) between the deletion and haplotypes B28 or A27 and estimation of the frequency of the deletion in different Nordic Red cattle populations. (PDF) [file pgen.1004049.s007.pdf]

Text S1: calling the deletion genotype based on total signal intensity, homozygosity, parentage conflicts and haplotype

Data

Total signal intensity. The total intensity data for 50K chip typing was available only for the recently genotyped animals. In total, 6012 animals (1312 Danish, 1605 Swedish and 3095 Finnish) had total signal intensity (log R ratio) for the five SNPs within the deletion.

Bovine50K genotypes. A total of 13,944 animals (2855 Danish, 4158 Swedish, 6894 Finnish, 16 Norwegian and 21 other) had genotypes and were phased (we know if they carry haplotype B28).

Method

Step 1: calling carriers based on genotypes and parentage conflict. We called the deletion status of all genotyped animals using information from homozygosity at the five SNPs encompassing the deletion and Mendelian parent-offspring incompatibilities (at one of the five SNPs). Individuals homozygous at the five SNPs and presenting a parentage conflict were declared carriers and individuals heterozygous for at least one SNP were declared non-carriers. The remaining individuals were set as 'unknown'.

Step 2: estimating the distribution of total signal intensity per SNP for carriers and non-carriers. Using the individuals declared carriers and non-carriers in Step 1, we obtained the distribution of log R ratio (**LRR**) values per SNP separately for carriers and non-carriers. The probability distribution was estimated for 50 bins of uniform width ranging from minimum to maximum LRR values. For each of the bin, we had the frequency (or the probability) to observe a LRR value within the bin given that the individual was a carrier (or a non-carrier).

Step 3: calling the deletion genotype from the total intensity data. After the distribution of LRR values conditional of the deletion genotype is estimated, we can call the deletion for any individual having signal intensity data (even without parentage conflict information). We can estimate the likelihood L_1 to observe the LRR values observed at the five SNP for a carrier:

$$L_1 = \prod_{i=1}^{5} P(LRR_i|carrier)$$

Similarly, we can compute the same likelihood L_2 for non-carriers:

$$L_2 = \prod_{i=1}^{5} P(LRR_i|noncarrier)$$

Where i is the SNP number, LRR_i is the observed log R ratio value observed at SNP i and $P(LRR_i|carrier)$ is the probability to observe the log R ratio at SNP i for a carrier (estimated in step 2).

The probability to be a carrier can then be estimated as the probability to observe the LRR values at the five SNP for a carrier divided by the probability to observe the LRR values at the five SNP (irrespective of the genotype at the deletion):

$$P(carrier) = \frac{L_1}{L_1 + L_2}$$

Step 4: estimating the linkage disequilibrium between haplotype B28 and deletion genotype. The haplotype B28 was estimated at marker position 20.866586 (minimizing homozygosity and extremely close to the deletion). We compute the squared-correlation between carriers of haplotype B28 (0 for non-carriers and 1 for carriers) and genotype and the deletion (0 for non-carriers and 1 for carriers) to estimate the linkage disequilibrium. Carriers and non-carriers of the deletion were individuals which were declared carrier or non-carrier in both Step 1 and Step 3. These genotypes were called high-confidence genotypes. Individuals with conflict between Step 1 and 3 were not used.

Results

Calling carriers based on genotypes and parentage conflict. Using the rules described above, 786 (177 Danish, 167 Swedish and 442 Finnish) were declared carriers and 3677 non-carriers (920 Danish,1055 Swedish and 1702 Finnish) among individuals with signal intensity data. All individuals with a parentage conflict were homozygous at the five SNPs.

Calling the deletion genotype from the total intensity data. Out of 6012 animals with intensity data, 261 Danish, 410 Swedish and 1031 Finnish were called carriers based on the signal intensity, corresponding to respectively 19.9%, 25.5% and 33.3% of carriers. These correspond to the frequency in the recent years since signal intensity is available for recently genotyped individuals.

Linkage disequilibrium between haplotype B28 and deletion genotype. Using a subset of 4456 individuals (1096 Danish, 1221 Swedish and 2139 Finnish) with deletion genotype called in step1 and in step3, without conflict between step 1 and step3 (4463 had both information and only 7 presented a conflict) and with haplotype information, it was estimated that the squared-correlation was equal to 0.96 (using haplotype A27, from the first phasing, the squared-correlation was equal to 0.93).

Frequency of carriers based on haplotype B28. Using individuals having information for signal intensity, haplotype frequencies estimated from haplotype B28 are 18.9%, 23.5% and 31.7% in Danish, Swedish and Finnish Red cattle, respectively. These values are close to those obtained with total intensity data are reported above. Using all the genotyped individuals, frequencies estimated from haplotype B28 are respectively 13.5%, 22.8% and 32.2% of carriers.
