# Supplementary material for: A 660-Kb Deletion with Antagonistic Effects on Fertility and Milk Production Segregates at High Frequency in Nordic Red Cattle: Additional Evidence for the Common Occurrence of Balancing Selection in Livestock
Source: PLoS Genet. 2014 Jan 2;10(1):e1004049. doi: 10.1371/journal.pgen.1004049 (PMC3879169; doi:10.1371/journal.pgen.1004049)
Supplement: Text S2 — Description of fertility traits. This document gives a brief description of all fertility traits used in the present study (including the number of genotyped individuals with records per trait). (PDF) [file pgen.1004049.s008.pdf]

Text S2. Description of fertility traits

Breeding values for the following traits are separately calculated for Heifers (with suffix h) and for cows (with suffix c) wherever applicable

(i) **Number of Insemination (AIS):** It is the number of insemination needed to get the cow/heifer into pregnancy; it reflects animal's ability to come to heat and to conceive.

(ii) **Interval from calving to first insemination (ICF):** The trait is defined only for cows and it is the interval in days between calving and the subsequent insemination. It reflects cow's ability to resume reproductive cycle after calving.

(iii) **Interval from First to Last insemination (IFL):** This is the number of days between the first and the last insemination a cow/heifer receives; it reflects the pregnancy rate and heat strength of an animal

(iv) **56-day Non return rate (NRR):** This trait indicates whether a cow/heifer receives a second insemination 56 days post first insemination. All cows/heifers not re-inseminated within 56 days post first insemination are considered pregnant. The trait reflects the pregnancy rate.

(v) **Heat strength (HST):** It is the ability of cow/heifer to show estrous, measured on subjective scale 1-5. The trait is recorded only in Sweden.

(vi) **Fertility Index (FI):** It is an index estimated from the sub-indices for AIS in cows and heifers, ICF in cows, IFL in cows and heifers combined by means their respective economic values. It reflects the genetic ability of the bull's daughter to show estrous and to conceive with an insemination.

The number of animals with EBV records for different fertility traits is tabulated below

	JER	HOL	RDCDNK	RDCSWE	RDCFIN	Total
**aisc**	1171	4067	891	1714	2240	10083
**aish**	1175	4071	893	1713	1972	9824
**icf**	1174	4070	891	1714	2242	10091
**iflc**	1173	4070	891	1714	2242	10090
**iflh**	1173	4070	892	1713	1969	9817
**nrrc**	1171	4070	891	1714	2240	10086
**nrrh**	1177	4071	893	1713	1974	9828
**frugti**	1177	4072	894	1714	2242	10099

The number of records for de-regressed proofs for fertility and production traits is given below.

	RDCDNK	RDCSWE	RDCFIN
aisc	776	1656	2165
aish	777	1664	2025
icf	775	1649	2156
iflc	777	1656	2166
iflh	777	1664	2103
nrrc	776	1650	2158
nrrh	777	1664	2141
milk	925	1494	2376
prot	925	1494	2376
fat	925	1494	2376
